# High Burden of Impetigo and Scabies in a Tropical Country

**DOI:** 10.1371/journal.pntd.0000467

**Published:** 2009-06-23

**Authors:** Andrew C. Steer, Adam W. J. Jenney, Joseph Kado, Michael R. Batzloff, Sophie La Vincente, Lepani Waqatakirewa, E. Kim Mulholland, Jonathan R. Carapetis

**Affiliations:** 1 Centre for International Child Health, University of Melbourne, Melbourne, Australia; 2 Fiji Ministry of Health, Suva, Fiji Islands; 3 Queensland Institute of Medical Research, Brisbane, Australia; 4 London School of Hygiene and Tropical Medicine, London, United Kingdom; 5 Menzies School of Health Research, Charles Darwin University, Darwin, Australia; Emory University, United States of America

## Abstract

**Background:**

Impetigo and scabies are endemic diseases in many tropical countries; however the epidemiology of these diseases is poorly understood in many areas, particularly in the Pacific.

**Methodology/Principal Findings:**

We conducted three epidemiological studies in 2006 and 2007 to determine the burden of disease due to impetigo and scabies in children in Fiji using simple and easily reproducible methodology. Two studies were performed in primary school children (one study was a cross-sectional study and the other a prospective cohort study over ten months) and one study was performed in infants (cross-sectional). The prevalence of active impetigo was 25.6% (95% CI 24.1–27.1) in primary school children and 12.2% (95% CI 9.3–15.6) in infants. The prevalence of scabies was 18.5% (95% CI 17.2–19.8) in primary school children and 14.0% (95% CI 10.8–17.2) in infants. The incidence density of active impetigo, group A streptococcal (GAS) impetigo, *Staphylococcus aureus* impetigo and scabies was 122, 80, 64 and 51 cases per 100 child-years respectively. Impetigo was strongly associated with scabies infestation (odds ratio, OR, 2.4, 95% CI 1.6–3.7) and was more common in Indigenous Fijian children when compared with children of other ethnicities (OR 3.6, 95% CI 2.7–4.7). The majority of cases of active impetigo in the children in our study were caused by GAS. *S. aureus* was also a common cause (57.4% in school aged children and 69% in infants).

**Conclusions/Significance:**

These data suggest that the impetigo and scabies disease burden in children in Fiji has been underestimated, and possibly other tropical developing countries in the Pacific. These diseases are more than benign nuisance diseases and consideration needs to be given to expanded public health initiatives to improve their control.

## Author Summary

Scabies and impetigo are often thought of as nuisance diseases, but have the potential to cause a great deal of morbidity and even mortality if infection becomes complicated. Accurate assessments of these diseases are lacking, particularly in tropical developing countries. We performed a series of studies in infants and primary school children in Fiji, a tropical developing country in the South Pacific. Impetigo was very common: more than a quarter of school-aged children and 12% of infants had active impetigo. Scabies was also very common affecting 18% of school children and 14% of infants. The group A streptococcus was the most common infective organism followed by *Staphylococcus aureus*. The size of the problem has been underestimated, particularly in the Pacific. It is time for more concerted public health efforts in controlling impetigo and scabies.

## Introduction

A recent review by the World Health Organization Department of Child and Adolescent Health and Development indicated that impetigo and scabies are endemic disease in many tropical and subtropical countries [1]. This review concluded that more data documenting the burden of skin disease in children are required at the community level because of gaps in the evidence, particularly relating to disease burden in infants and the reporting of incidence data. The authors of the review also concluded that comparisons between studies of childhood skin disease are hindered by a lack of uniform study methodology, particularly standardized case definitions and the routine inclusion of microbiologic data.

This current study is a comprehensive investigation into the epidemiology of scabies and impetigo in Fiji. We followed an epidemiologic protocol developed in 2005 by a World Health Organization / US National Institutes of Health working group that is currently being prepared for publication (personal communication, F Rubin, National Institutes of Health), with some modifications to suit the Fiji context. We used case definitions that are simple and provide easily reproducible results. Our study focused on school-aged children and infants, and provides prevalence, incidence and microbiologic data.

## Methods

### Objectives and study design

We conducted three epidemiologic studies in 2006 and 2007 in order to define the burden of disease of impetigo and scabies in children in Fiji. The first was a cross-sectional survey in a large number of primary school children, the second was a prospective ten month study in a smaller cohort of school children, and the third was a cross-sectional survey of infants.

### Setting

Fiji is a nation of approximately 330 islands located in the Western Pacific. It has a population of 827,900 people comprising of two major racial groups of Indigenous Fijians (57.3%) and Indo-Fijians (37.6%) [2]. Fiji is ranked 90 out of 177 nations on the United Nations Development Programme Human Development Index. It has a GDP per capita of USD6,066 and an infant mortality rate of 16.8 per 1000 [3],[4]. Approximately 49% of the population lives in rural areas [2]. This study was conducted in primary schools and child-health centres in the Central Division of Fiji (population 340,843 people, 75,462 aged 5–14 years) [2]. Primary schools in Fiji extend from class one (five years of age) to class eight (14 years of age).

#### Study one

This study was a cross sectional prevalence survey of school children aged 5–15 years from 21 schools in the Central Division of Fiji. These schools were chosen by random sampling, with stratification for geographic location (urban versus rural) and for ethnicity. Microbiologic specimens were not collected in this study.

#### Study two

This was a prospective cohort study performed in school children aged five to 15 years in three of the 21 schools included in Study One. These three schools were selected a priori with the aim of having a closely-followed prospective cohort that was commensurate to the Indigenous Fijian/Indo-Fijian and urban/rural proportions of Study One. As such, School A and school B were Indigenous Fijian schools located in a rural area, whilst school C was a larger predominantly Indo-Fijian school located in Fiji's capital city, Suva. Each school was visited six times over a ten month period, at two monthly intervals. The examinations undertaken as part of Study One served as the first visit in these three schools. Microbiological specimens were collected at each visit in this study.

#### Study three

This study was a cross-sectional survey of infants attending for routine checks at two maternal and child health care clinics (one rural clinic in proximity to Schools A and B, and one urban clinic in proximity to School C). Microbiological confirmation of GAS and S. aureus impetigo was undertaken in this study.

### Study procedures and case definitions

The protocol and case definitions were applied consistently across all three studies. Children were examined for the clinical presence of impetigo and scabies, with the initial examination restricted to the arms, legs, head and neck to avoid embarrassment. Any children with severe scabies or impetigo also had an examination of the abdomen and back; children did not have an examination of the buttocks, groin or chest, except in the case of infants in whom scabies and impetigo lesions are often present in atypical areas [5]. Research staff were trained in dermatologic clinical examination, including the clinical diagnosis of impetigo and scabies, and were supervised by a paediatrician. Active impetigo was defined as any crusted ulcer or vesiculopustular skin eruption, whilst old impetigo was defined as any dry skin lesion. Active impetigo was classified as being mild (less than five lesions), moderate (between five and 20 lesions) or severe (more than 20 lesions). Where microbiologic data was available, a case of GAS impetigo was defined as a child with one or more active impetigo lesions from which GAS was grown from culture of at least one lesion; *Staphylococcus aureus* impetigo was defined similarly when *S. aureus* was cultured. Scabies was defined on the basis of typical clinical findings, that is, inflammatory papules with a typical distribution that were pruritic, without the use of microscopy or dermatoscopy [6]. Scabies lesions were classified according to presence of bacterial superinfection (i.e. ‘scabies’ or ‘infected scabies’).

#### Treatment

We referred children identified with moderate or severe impetigo, purulent impetigo or scabies to their local health clinic for treatment using local guidelines which include oral flucloxacillin for impetigo and topical benzoyl benzoate for scabies [7]. This strategy was agreed upon following discussion with the Fiji Ministry of Health, the relevant health clinics and the ethics committee in Fiji. Information sheets about skin sores and scabies written in plain language were provided to the children and their parents.

#### Laboratory methods

In Study Two and Study Three, children identified with vesiculopustular impetigo had a maximum of two skin swabs taken of impetigo lesions. If two swabs were taken, they were taken from as distant anatomical sites as possible. The skin was not cleaned prior to taking a swab. Purulent lesions were given priority, with swabs of these lesions taken by rolling the swab over the purulent lesion. If a swab of a crusted impetigo lesion was taken, this was done by gently lifting the crust and rolling the swab over the base of the lesion. Skin sore swabs were transported in a sealed bag containing dessicant and inside a cold box as described previously [8], and were plated onto sheep blood within six hours. Following incubation at 37 degrees C in 5% CO2 for up to 24 hours, plates were checked for beta-hemolytic colonies and the predominant colony on each plate was sub-cultured onto sheep blood agar and re-grown for Lancefield grouping (Oxoid, Cambridge, UK). Plates were also checked for growth of S. aureus based upon colony morphology and confirmed using Staphaurex (Remel Inc, Kent, UK).

### Ethical approval

Ethical approval was obtained from the Fiji National Research Ethics Review Committee, the Fiji National Health Research Committee, the University of Melbourne Human Research Ethics Review Committee, the Queensland Institute of Medical Research Human Research Ethics Review Committee and the Menzies School of Health Research Human Research Ethics Review Committee. Before commencing the study we conducted visits to the schools to explain the study to students, parents and teachers in open forums. Information sheets in Fijian and English were provided to families. Children were only enrolled if written consent from a parent or guardian was obtained, and in the case of children aged ten years or older if written assent was also obtained.

### Statistical analysis

For the cross-sectional studies, we calculated prevalence of active impetigo and scabies with binomial 95% confidence intervals (CI). Logistic regression was used to assess the association between demographic and clinical factors and the outcome of interest (presence of active impetigo and scabies). The models accounted for clustering by school and included the following variables: gender, age, ethnicity, location of school (urban or rural), body mass index-for-age and either impetigo or scabies.

For the prospective study we calculated point prevalence at each visit and used a two sample test of proportion to test differences between prevalence at each visit. We calculated person-time incidence rates (incidence density), which allowed for calculation of an incidence rate even if visits were missed. The numerator was the number of incident cases; an incident case of active impetigo being defined as a child with an active lesion who did not have any active lesions at the previous visit (this could occur more than once for each child). The denominator was the disease-free time at risk which was the total period of time between consecutive disease-free visits. We also calculated cumulative incidence (the proportion of children that became affected by disease during the study period). The denominator was the number of children that had complete follow-up and were disease-free at the start of the study, and the numerator was the number of these children that developed a new episode of disease during the study period. The same principles for defining incident cases and calculating incidence rates were applied to GAS impetigo, *S. aureus* impetigo and scabies. When analysing demographic and clinical associations in the prospective study we used incidence rate ratio calculations based upon incidence density calculations. The statistical package STATA version 10.0 (Stata Corporation, College Station, Texas, USA) was used to analyse the data.

## Results

### Study one

Of 5562 children invited to participate in this study, 3500 were enrolled (61.9%), and of these, 38 children were excluded from further analysis because of their age, making a total of 3462 children aged five to 15 years in the study sample. The median age of the sample was 10.2 years (interquartile range 8–12.2 years). There were 2332 Indigenous Fijian Children (67.4%), 969 Indo-Fijian children (28.0%) and 161 children of other races (4.6%). There were 1541 children (44.5%) that attended a school located in a rural area.

Table 1 summarises prevalence figures for impetigo and scabies. Impetigo was found more commonly on the lower extremities than the upper limbs (56.0% lower limbs only, 11.9% upper limb only, 32.1% both upper and lower limbs, p<0.001), whilst scabies was found more commonly on the upper limb (65.8% upper limbs only, 4.1% lower limb only, 30.1% both upper and lower limbs, p<0.001).

**Table 1:** Prevalence of scabies and impetigo in 3462 primary school children (CI: confidence interval).

			Total			5–8 years			9–11 years			12–15 years		
			n	Prevalence (%)	95% CI	n	Prevalence (%)	95% CI	n	Prevalence (%)	95% CI	n	Prevalence (%)	95% CI
Impetigo	All		1259	36.4	34.8–38.0	372	42.9	39.6–46.2	449	37.9	35.1–40.7	438	31.1	28.6–33.5
	Active		885	25.6	24.1–27.1	268	30.9	27.8–34.1	329	27.8	25.2–30.4	288	20.4	18.3–22.6
		Mild	640	18.5	17.2–19.8	195	22.5	19.7–25.3	221	18.6	16.4–20.9	244	15.9	14.0–17.8
		Moderate	182	5.3	4.5–6.1	48	5.5	4.0–7.1	82	6.9	5.5–8.4	52	3.7	2.7–4.7
		Severe	63	1.8	1.4–2.3	25	2.9	1.8–4.0	26	2.2	1.4–3.0	12	0.9	0.4–1.3
Scabies	All		640	18.5	17.2–19.8	213	24.6	21.7–27.6	213	18.0	15.8–20.3	214	15.2	13.3–17.2
	Infected		191	5.5	4.5–6.3	65	7.5	5.7–9.3	67	5.7	4.3–7.0	59	4.2	3.1–5.2

There was significant variation across the 21 schools; the highest prevalence of active impetigo by school was 55.8%, and the lowest was 7.1% with a median of 24.5% , and the highest prevalence of scabies by school was 42.9% and the lowest was 4.8% with a median of 12.9%. This variation appeared to be due to ethnicity and the presence of scabies by school; logistic regression analysis of demographic factors revealed Indigenous Fijian race and the presence of scabies to be significantly associated with active impetigo (Table 2). Children aged six years were at greatest risk of active impetigo and scabies, with a prevalence of active impetigo and scabies in this group of 33.9% (95% CI 29–39.1) and 26.6% (95% CI 22.0–21.5) respectively.

**Table 2:** Tests of association for the prevalence of active impetigo and scabies in children aged 5–15 years at 21 primary schools Fiji.

Factor		Total	Active impetigo			Scabies		
			n	Prevalence as percentage (95% CI)	Odds ratio* (95% CI)	n	Prevalence as percentage (95% CI)	Odds ratio* (95% CI)
**Gender**	Male	1692	532	31.4 (29.2–33.7)	1.9 (1.6–2.2)	326	19.3 (17.4–21.2)	1.0 (0.8–1.2)
	Female	1770	353	19.9 (18.1–21.9)		314	17.7 (16.0–19.6)	
**Ethnicity**	Fijian	2332	770	33.0 (31.1–35.0)	3.6 (2.7–4.7)	587	25.2 (23.4–27.0)	4.8 (2.5–9.4)
	Other	1130	115	10.2 (8.5–12.1)		53	13.5 (11.6–15.7)	
**Location of school**	Rural	1541	481	31.2 (28.9–33.6)	1.1 (0.6–2.0)	376	24.4 (22.3–26.6)	1.3 (0.7–2.3)
	Urban	1921	404	21 (19.2–22.9)		264	13.7 (12.2–15.4)	
**Age**	5–8 years	867	268	30.9 (27.8–34.1)	0.91 (0.87–0.94)†	213	24.6 (21.7–27.6)	0.91 (0.87–0.96)†
	9–11 year	1185	329	27.8 (25.2–30.4)		213	18.0 (15.8–20.3)	
	12–15 years	1410	288	20.4 (18.3–22.6)		214	15.2 (13.3–17.2)	
**Active impetigo**	Impetigo	885	-	-	-	300	33.9 (30.8–37.1)	2.2 (1.5–3.2)
	No impetigo	2577	-	-		340	13.2 (11.9–14.6)	
**Scabies**	Scabies	640	300	46.9 (43.0–50.8)	2.4 (1.6–3.7)	-	-	-
	No scabies	2822	585	20.7 (19.2–22.3)		-	-	
**Overall**		3462	885	25.6 (24.1–27.1)		640	18.5 (17.2–19.8)	

*: Odds ratios were calculated using a logistic regression model.

**†:** Odds ratios for age were calculated using age as a continuous variable in the logistic regression model.

### Study two

Of the 685 children eligible for the study, 457 children (66.7%) were enrolled; 80 children at school A (enrolment rate 96.4%), 175 at school B (80.6%) and 202 at school C (53.2%). There were 322 Indigenous Fijian Children (70.5% of sample), 99 Indo-Fijian children (21.7%) and 36 children of other races (7.8%). Just over half of the enrolled children attended one of the two rural schools (n = 255, 55.8%) and all of these children were Indigenous Fijian. The median age of the sample was 9.9 years (interquartile range 7.9–12 years). All 457 children enrolled into the study were seen at the initial visit, and of these, 378 children were seen at all six visits; 42 children were seen on five occasions, six children on four occasions, three children on three occasions, six children on two occasions visits and 22 children were seen only at the first visit. In total there were 2545 examinations performed, and a total of 114667 child-days of observation (314 years).

#### Prevalence

**Figure 1:**
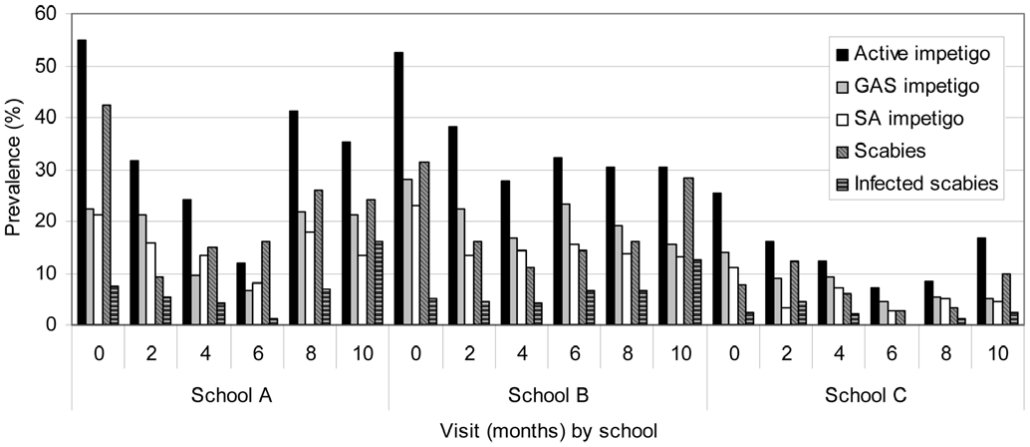
Prevalence of skin disease by school and by visit over a period of 10 months at three primary schools in Fiji.

Of the 457 children seen at the first visit, 187 had active impetigo (prevalence 40.9%, 95% CI 36.4–45.6), 95 had GAS impetigo (prevalence 20.8%, 95% CI 17.2–24.8) and 105 had scabies (prevalence 23.0%, 95% CI 19.2–27.1). The prevalence of impetigo and scabies varied by school and by visit (Figure 1). The prevalence of active impetigo reduced by 67.7% at the second visit to a prevalence of 27.7% (95% CI 23.6–32.2, p<0.0001) and this significant reduction was sustained throughout the period of the study (prevalence at last visit 25.7%, 95% CI 21.5–30.3, p<0.0001). The prevalence of GAS impetigo decreased at the second visit to a prevalence of 16.6% (95% CI 13.2–20.4, p = 0.11), and although this reduction was not significant, further reductions occurred so that the prevalence at the final visit fell by 50.9% when compared with the first visit (prevalence 10.6%, 95% CI 7.7–14.0, p<0.001). The prevalence of scabies reduced by 57.8% at the second visit to a prevalence of 13.3% (95% CI 10.2–16.9, p<0.001), however this reduction was not maintained throughout the study period (prevalence at last visit 20.1%, 95% CI 16.3–24.4, p = 0.3).

#### Incidence density and cumulative incidence

Table 3 summarizes incidence density data for active impetigo, GAS impetigo, S. aureus impetigo and scabies. Of the 378 children that had full follow-up (that is, 6 visits), there were 219 (57.9%) that were free of active impetigo at the start of the study. Of these, 104 children (47.5%) had at least one new episode of during the course of the study. For GAS impetigo, S. aureus impetigo and scabies, 36.7%, 32.9% and 24.6% of disease free children at the start of the study had a new episode of disease respectively.

**Table 3:** Incidence density of new cases of active impetigo, group A streptococcal (GAS) impetigo, *Staphylococcus aureus* (SA) impetigo and scabies in three Fijian schools.

	Disease free days	New cases	Incidence per 100 child-years	95% confidence interval
Active impetigo	83,132	277	122	108–137
GAS impetigo	96,447	212	80	75–85
SA impetigo	100,444	175	64	55–74
Scabies	97,580	136	51	43–60

Because scabies is characterized by ongoing infestation we analysed data relating to the number of children that had scabies found at more than one visit. Of the 378 children that had full follow-up, there were nine who had scabies at all six visits, nine at five visits, 10 at four visits, 16 at three visits and 36 at two visits.

#### Demographic analysis

Analysis of demographic data revealed that incidence rates of GAS impetigo and scabies were significantly higher in Indigenous Fijian children (incidence rate ratio for GAS impetigo 4.5, 95% CI 2.9–7.8, and incidence rate ratio for scabies 3.4, 95% 2.0–6.2). Children aged 5–9 years had a higher incidence of GAS impetigo and scabies than children aged 10–14 years (incidence rate ratio for GAS impetigo 1.6, 95% CI 1.2–2.1, and incidence rate ratio for scabies 2.2, 95% CI 1.5–3.2).

#### Microbiology

A total of 563 swabs were taken from 455 children (108 children had two swabs taken) and there was bacterial growth from 522 swabs (92.7%). There was growth of beta-hemolytic streptococci from 491 swabs (87.2%). GAS was isolated from 449 swabs (79.8% of all swabs), GGS from 28 swabs (5.0%), GCS from 11 swabs (2.0%), GBS from one swab, and two isolates were not able to be characterized by carbohydrate grouping. S. aureus was isolated from 323 swabs (57.4%) and co-existed with beta-hemolytic streptococci in 292 swabs (51.9%), of which 265 were GAS (47.1% of total).

Of the 108 children that had two swabs taken, 85 had BHS isolated from both swabs, of which 77 children had GAS isolated from both swabs, one child had GGS isolated from both swabs, whilst eight children had a GAS isolated from one swab and a GGS isolated from the other swab. Of the 77 children with GAS isolated from both swabs, 60 children were found to have the same *emm* type of GAS in both swabs.

### Study three

There were 451 infants enrolled in this study, divided between the rural and urban clinics (rural clinic n = 226, urban clinic n = 225). The median age of the sample was 20 weeks of age (interquartile range 8–37 weeks), and 265 children were aged six months or less (58.8%). There were 304 Indigenous Fijian Children (67.4%), 111 Indo-Fijian children (24.6%) and 36 children of other races (8.0%).

Table 4 summarises prevalence data for impetigo and scabies in infants. There was no clear association between impetigo and distribution on the upper or lower limbs (37% lower limbs only, 13% upper limb only, 50% both upper and lower limbs, p = 0.15). Scabies was more commonly found on the lower extremities (39.7% lower limbs only, 9.5% upper limb only, 50.8% both upper and lower limbs, p = 0.04). In the logistic regression model there was a clear association between GAS impetigo and scabies (OR 36.9, 95% CI 16.9–80.7).

**Table 4:** Prevalence of scabies and impetigo in 451 infants (CI: confidence interval).

			Total			0–6 months			7–12 years		
			n	Prevalence (%)	95% CI	n	Prevalence (%)	95% CI	n	Prevalence (%)	95% CI
Impetigo	All		60	13.3	10.3–16.8	16	6.0	3.2–8.9	44	23.7	17.5–29.8
	Active		55	12.2	9.3–15.6	13	4.9	2.3–7.5	42	22.6	16.5–28.6
		Mild	38	8.4	6.0–11.4	11	4.2	1.7–6.6	27	14.5	9.5–19.6
		Moderate	8	1.8	0.8–3.5	1	0.4	0–1.1	7	3.8	1.0–6.5
		Severe	9	2.0	0.8–3.8	1	0.4	0–1.1	8	4.3	1.4–7.2
Scabies	All		63	14.0	10.7–17.2	24	9.1	5.6–12.5	39	21.0	15.1–26.9
	Infected		36	8.0	5.5–10.5	8	3.0	0.9–5.1	28	15.1	9.9–20.2

#### Microbiology

A total of 61 swabs were taken from 50 infants (11 infants had two swabs taken), and there was bacterial growth from 52 swabs (85%). There was growth of beta-hemolytic streptococci from 41 swabs (67%), and GAS was isolated from 30 swabs (49% of total number of swabs), GGS from eight swabs (13%), GCS from two swabs and GBS from one swab. S. aureus was isolated from 42 swabs (69%) and co-existed with beta-hemolytic streptococci in 31 swabs (51%), of which 23 were GAS (38% of total).

## Discussion

Our study indicates that there is a large burden of skin disease in children in Fiji. Over one quarter of school aged children in our cross-sectional study had active impetigo at any given time and almost half of impetigo-free children in our prospective cohort study experienced a new episode of active impetigo during the ten month study period. Nearly one-third of scabies-free children in the cohort experienced a new episode of scabies during the study period. Impetigo and scabies were also highly prevalent diseases in the infants observed in our study, although less common than in children. The majority of cases of active impetigo in the children in our study were caused by GAS (79.8% in school aged children, and 49% in infants), although *S. aureus* was also a common cause (57.4% in school aged children and 69% in infants).

A recent review of the prevalence of childhood skin diseases in developing tropical and subtropical countries concluded that the prevalence of impetigo is commonly in the range of 5–10% and the prevalence of scabies is in the range of 1–2% [1]. Many of the studies included in this review were from sub-Saharan Africa. Studies from the Pacific, including ours, suggest that the prevalence of impetigo and scabies is significantly higher in the Pacific region [9],[10],[11],[12]. The reason for this is not clear. A significant finding in our study was the higher risk of GAS impetigo and scabies in Indigenous Fijian children.

We have previously noted higher rates of GAS disease in Indigenous Fijians, including invasive GAS disease and rheumatic heart disease [13],[14]. Other investigators have noted higher rates of other Gram-positive infections, including radiographically proven pneumonia [15].

We used a simple method to calculate incidence density and found a very high incidence density of new cases active impetigo and scabies. It is likely that our figures are in fact an underestimate of the true incidence for three reasons. First, we may have missed new cases of impetigo and scabies that erupted and then healed in between the two-monthly study visits. A study in Mali estimated that 41% and 53% of cases of impetigo and scabies lasted for less than one month respectively [16]. Second, we required a child to be disease-free at the previous visit before a case could be counted as a new case; we therefore did not count new lesions in children with existing lesions as incident cases – to detect these cases would require more intensive surveillance techniques such as interval photography. Third, the method of clinical examination in this study may have underestimated prevalence because the entire body was not always examined. There were also other limitations to our study including potential sources of bias. There was the potential for participation bias because of differing enrolment rates between schools and between studies, and there was also the potential for inter-rater variability and bias because of multiple observers, although we attempted to standardize the clinical examination methodology as much as possible.

Impetigo and scabies have been considered public health problems in developing countries for decades [16],[17], however there has been little progress in their control on a global scale [1],[18]. It is estimated that there are more than 111 million prevalent cases of GAS pyoderma globally [19]. Although often considered as a benign disease, impetigo can lead to more serious illnesses including cellulitis and abscess via local spread, bacteremia and sepsis following haematogenous invasion, as well as the non-suppurative sequelae of acute post-streptococcal glomerulonephritis and possibly acute rheumatic fever [20]. The highest incidence rates of invasive GAS disease have been described in tropical developing countries and impetigo has been identified as an important portal of entry [13],[21],[22]. The mortality rate from invasive GAS disease in these settings is high; in Fiji the all-ages case fatality rate is over 25% [13]. Post-streptococcal glomerulonephritis in tropical regions almost always follows impetigo GAS infection rather than GAS pharyngitis as it does in industrialised countries [23],[24],[25]. There is evidence to suggest that acute post-streptococcal glomerulonephritis may contribute to chronic renal impairment in adult life [26]. Scabies on its own can cause significant clinical impact, most notably sleep disturbance that occurs in up to 70% of cases [5]. In addition to their clinical impact, impetigo and scabies also cause a considerable financial burden to individuals, families and health services. From the limited published data, impetigo and scabies account for between 12.3% and 23.7% of primary health care centre presentations in tropical countries, placing a significant drain on these services [1]. Impetigo and scabies leads to absence from school and costly treatment. In a study in Mexico, the average period of absence from school for scabies was 8 days and for pyoderma 15 days, and the average cost of treatment for scabies and impetigo was US$24 and US$52 respectively [1],[27].

Given the clinical and economic burden of impetigo and scabies, and the sheer volume of cases in tropical developing countries, there have been renewed appeals for an organized global effort to control these diseases [1]. However, in many countries where impetigo and scabies are common, there are competing health problems that carry higher morbidity and mortality. Therefore, it has been suggested that control measures be simple, practical, of low cost and commensurate with the level of the priority of the problem in the local context [1]. We have recently validated an algorithm for the identification and treatment of common childhood skin conditions that can be incorporated into existing Integrated Management of Childhood Illness programs [28]. This algorithm is designed to improve case management of childhood skin diseases at the primary health care level, with minimal need for additional infrastructure and training.

In countries such as Fiji that have been able to significantly reduce high mortality diseases and in which skin disease is common, it may be appropriate to consider initiatives aimed at controlling impetigo and scabies on a broader public health level. Whilst mass drug treatment with scabicides can produce short-term reduction in scabies disease burden [29], most experts believe that a sustained reduction requires an integrated approach that includes the following elements: public health education, improved case management, improved drug supply and potentially improvements in personal hygiene practices [18],[30]. Intervention studies in the Pacific region aimed solely at scabies control have been shown to achieve their aim of controlling scabies with a flow-on effect on scabies-associated impetigo, but they have had less effect on non-scabies impetigo [10],[31]. The finding of a large burden of impetigo independent of scabies in school-aged children in our study suggests that the case may be the same in Fiji [9],[11]. Further research into practicable, effective and sustainable control measures is required.

A GAS vaccine could also be an important advance in primary prevention of impetigo. However, major questions remain about a GAS vaccine designed to prevent skin disease. First, little is known about the mechanism of immunoprotection against impetigo [32],[33], because group A streptococcal vaccine research has largely focused on protection against pharyngitis and invasive disease. Second, it is not clear what effect a GAS vaccine would have on *S. aureus* in impetigo lesions. As we have shown, *S. aureus* is a common infecting agent in impetigo, and *S. aureus* and GAS can co-exist in individual lesions. It is possible that circulating GAS strains removed by a vaccine would only be replaced by *S. aureus* strains that are currently the victims of competitive inhibition by GAS. Third, the design of a GAS vaccine against skin disease would need to be specifically tailored to cover common types of GAS that cause impetigo.

Our study confirms that impetigo and scabies are endemic diseases in Fiji causing a substantial disease burden, consistent with findings from around the region. These findings, coupled with the potential for clinical complications and a heavy socio-economic impact, suggest that it is time for more concerted action against these diseases in Fiji and other tropical developing nations.

## Supporting Information

Checklist S1 STROBE Checklist
(0.10 MB DOC)
